# The clinical, myopathological, and genetic analysis of 155 Chinese mitochondrial ophthalmoplegia patients with mitochondrial DNA single large deletions

**DOI:** 10.1002/mgg3.2328

**Published:** 2023-11-28

**Authors:** Yang Zhao, Yue Hou, Xutong Zhao, Tongling Liufu, Meng Yu, Wei Zhang, Zhiying Xie, Victor Wei Zhang, Yun Yuan, Zhaoxia Wang

**Affiliations:** ^1^ Department of Neurology Peking University First Hospital Beijing China; ^2^ Beijing Key Laboratory of Neurovascular Disease Discovery Beijing China; ^3^ Department of Geriatrics Peking University First Hospital Beijing China; ^4^ Department of Neurology Beijing Jishuitan Hospital Beijing China; ^5^ AmCare Genomics Lab Guangzhou China

**Keywords:** chronic progressive external ophthalmoplegia, Kearns–Sayre syndrome, mitochondrial DNA, single large‐scale deletion

## Abstract

**Background:**

Progressive external ophthalmoplegia (PEO) is a common subtype of mitochondrial encephalomyopathy.

**Objective:**

The study aimed to investigate the relationship between mitochondrial DNA (mtDNA) abnormalities, muscle pathology, and clinical manifestations in Chinese patients with single large‐scale mtDNA deletion presenting with PEO.

**Methods:**

This is a retrospective single‐center study. Patients with PEO who had a single large deletion in mitochondrial DNA were included in this study. The associations were analyzed between mtDNA deletion patterns, myopathological changes, and clinical characteristics.

**Results:**

In total, 155 patients with mitochondrial PEO carrying single large‐scale mtDNA mutations were enrolled, including 137 chronic progressive external ophthalmoplegia (CPEO) and 18 Kearns–Sayre syndrome (KSS) patients. The onset ages were 9.61 ± 4.12 in KSS and 20.15 ± 9.06 in CPEO. The mtDNA deletions ranged from 2225 bp to 9131 bp, with m.8470_13446del being the most common. The KSS group showed longer deletions than the CPEO group (*p* = 0.004). Additionally, a higher number of deleted genes encoding respiratory chain complex subunits (*p* = 0.001) and tRNA genes (*p* = 0.009) were also observed in the KSS group. A weak negative correlation between the mtDNA deletion size and ages of onset (*p* < 0.001, *r* = −0.369) was observed. The proportion of ragged red fibers, ragged blue fibers, and cytochrome *c* negative fibers did not correlate significantly with onset ages (*p* > 0.05). However, a higher percentage of abnormal muscle fibers corresponds to an increased prevalence of exercise intolerance, limb muscle weakness, dysphagia, and cerebellar ataxia.

**Conclusion:**

We reported a large Chinese cohort consisting of mitochondrial PEO patients with single large‐scale mtDNA deletions. Our results demonstrated that the length and locations of mtDNA deletions may influence onset ages and clinical phenotypes. The severity of muscle pathology could not only indicate diagnosis but also may be associated with clinical manifestations beyond the extraocular muscles.

## Graphical abstract

Figure A illustrates the ROC curve depicting the relationship between the age of onset and the disease diagnosis. The AUC for distinguishing CPEO and KSS by ages of onset was 0.874, having a maximum Youden index of 0.594, resulting in an optimum cut‐off point of 15.5 years. Figure B displays a moderate negative correlation between the mtDNA deletion size and onset ages (correlation coefficient *r* = −0.369).

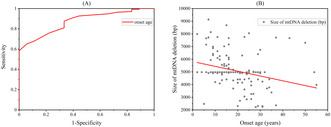

## 1 INTRODUCTION

Mitochondria are widely present in nucleated cells and can produce ATP as well as a variety of biosynthetic intermediates while playing a significant role in apoptosis and autophagy (Nunnari & Suomalainen, 2012). Unlike other organelles, the mitochondrion contains its own genome, a mitochondrial DNA (mtDNA), which has 16,569 base pairs (bp) in size containing 37 genes encoding 13 electron transport chain (ETC) complex subunit proteins, 22 transfer RNAs (tRNAs), and 2 ribosomal RNA (rRNAs) (El‐Hattab et al., 2017). Since its replication depends on nuclear genes such as *POLG* (OMIM accession number: *174763) and *TWNK* (OMIM accession number: *606075) (Paramasivam et al., 2016; Rahman & Copeland, 2019), abnormalities in either nuclear DNA (nDNA) or mtDNA can lead to mitochondrial dysfunction and consequently mitochondrial diseases. Mitochondrial dysfunction is the primary pathogenic mechanism of mitochondrial disease by affecting the cellular oxidative phosphorylation process, resulting in impaired cellular energy supply and thus tissue or organ damage. The central nervous system, cardiac and skeletal muscles, liver, kidney, and endocrine system are more susceptible due to their high energy consumption (El‐Hattab et al., 2017).

Mitochondrial disease has several subtypes with different pathogenic mechanisms. Common subtypes such as mitochondrial encephalomyopathy, lactic acidosis, and stroke‐like episodes (MELAS), and myoclonic epilepsy with ragged red fibers (MERRF) are characterized by point mutations in mtDNA (Finsterer et al., 2018; Tetsuka et al., 2021), while Kearns–Sayre syndrome (KSS) and chronic progressive external ophthalmoplegia (CPEO) are commonly caused by large deletions in mtDNA (Grady et al., 2014).

CPEO is characterized by the progressive restriction of eye movements, often with ptosis, and when combined with other muscle involvement and systemic symptoms, it is called “CPEO plus” (Drachman, 1968). KSS is a progressive multisystem disorder with onset before age 20 years characterized by pigmentary retinopathy, CPEO, and cardiac conduction abnormalities. Additional features can include cerebrospinal fluid (CSF) protein of more than 100 mg/dL, cerebellar ataxia, short stature, deafness, dementia, and endocrine abnormalities (Kearns & Sayre, 1958). Compared to CPEO plus, KSS patients have more severe muscle involvement and overall have a worse prognosis. While a small portion of CPEO is associated with mtDNA point mutation (7%), or nuclear genes (26%), at least 63% of CPEO patients were associated with single large‐scale deletions of mtDNA (Heighton, Brady, Sadikovic, et al., 2019; Rodriguez‐Lopez et al., 2020), Previous research has yielded inconsistent findings concerning the association between mitochondrial genetics and clinical manifestation. Specifically, the size of mtDNA deletions has been reported to exhibit varying levels of its predictability concerning the age of onset, clinical phenotype, and severity of symptoms (Grady et al., 2014), or only predicts the age of onset (López‐Gallardo et al., 2009), or is independent of the age of onset (Anteneová et al., 2020) and clinical phenotype (Moraes et al., 1989). The divergent conclusions mentioned above hinder the establishment of a comprehensive understanding of this particular cluster of diseases.

In this study, we retrospectively collected the clinical, myopathological, and genetic data from a large group of Chinese PEO patients with single large mtDNA deletions, aiming to investigate the relationship between mtDNA abnormalities, muscle lesions, and clinical manifestations.

## 2 METHOD

### 2.1 Editorial policies and ethical considerations

Our study complied with the ethical guidelines and was conducted under the World Medical Association Declaration of Helsinki. This study was approved by the Ethics Committee of Peking University First Hospital (2019–181).

### 2.2 Patient enrollment

Between 1996 and 2022, there were 155 patients diagnosed with mitochondrial PEO by clinical and pathological examination at the Department of Neurology at Peking University First Hospital. Only those PEO patients who underwent genetic tests were identified as carrying single large mtDNA deletions were recruited. Medical records were reviewed for demographic data, clinical manifestations, laboratory data, muscle pathology features, and data on mtDNA deletions. All patients gave informed consent before muscle biopsy or mtDNA analysis.

### 2.3 Pathology analysis

The muscle tissue specimens were pre‐cooled with isopentane, frozen, and fixed in liquid nitrogen, and cryosections were performed with a slice thickness of 6 μm. Routine histological and enzymatic histochemical staining was performed, including hematoxylin and eosin (H&E) staining, modified Gomori trichrome (mGT) staining, nicotinamide adenine dinucleotide‐tetrazolium reductase (NADH‐TR) staining, succinate dehydrogenase (SDH) staining, cytochrome *c* oxidase (COX), and succinate dehydrogenase (COX/SDH) double staining. All the sections were scanned as digital sections by Hamamatsu NanoZoomer and re‐analyzed for this study by two experienced pathologists (YY and ZW). Three high‐magnification fields of view (200x) were randomly selected for each digital section, and the number of both myofibers and abnormal myofibers, including ragged red fibers (RRFs), ragged blue fibers (RBFs), and COX‐negative fibers, were tallied in the selected fields. The proportion of abnormal myofibers was calculated subsequently.

### 2.4 Genetic analysis

DNA was extracted from patients' muscle tissues, and mtDNA deletions were detected by Southern blot (6 patients) or long‐range polymerase chain reaction (LR‐PCR, 149 patients). The specific deletion endpoints were determined by Sanger sequencing of short‐cycle PCR products (121 patients) or next‐generation sequencing (NGS, 34 patients) (Grady et al., 2014; Hou et al., 2022). This study examined the MT‐CO genes, including MT‐CO1, MT‐CO2, and MT‐CO3, and the MT‐CYB genes present in the deletion fragments. Their reference sequences and version numbers in GenBank were as follows: the accession numbers for MT‐CO1 (NC_012920 REGION: 5904..7445), MT‐CO2 (NC_012920 REGION: 7586..8269), MT‐CO3 (NC_012920 REGION: 9207..9990), and MT‐CYB (NC_012920 REGION: 14747..15887) were all version NC_012920.1.

### 2.5 Statistical analysis

Continuous variables were summarized using means ± standard deviations for normally distributed data, and medians (interquartile ranges, IQR) for non‐normally distributed data or ordinal data. Two‐sample independent t‐tests were used for group comparisons by continuous variables that conform to a normal distribution. The Mann–Whitney *U* test was used for comparisons between two groups that used continuous variables that did not follow a normal distribution. The Kruskal–Wallis test was used to compare multiple groups with continuous variables that did not follow a normal distribution. The chi‐square test was used to count data or rank data when the total sample size *n* ≥ 40 and all theoretical frequencies *T* ≥ 5. If the condition of the chi‐square test was not satisfied, the Fisher's exact probability method test was used. Spearman correlation analysis was used to analyze the correlation of two variables that do not obey a normal distribution. A *p* value < 0.05 was considered statistically significant. Data were analyzed with IBM SPSS Statistics V.27.0.

## 3 RESULTS

### 3.1 Clinical data

**FIGURE 1:**
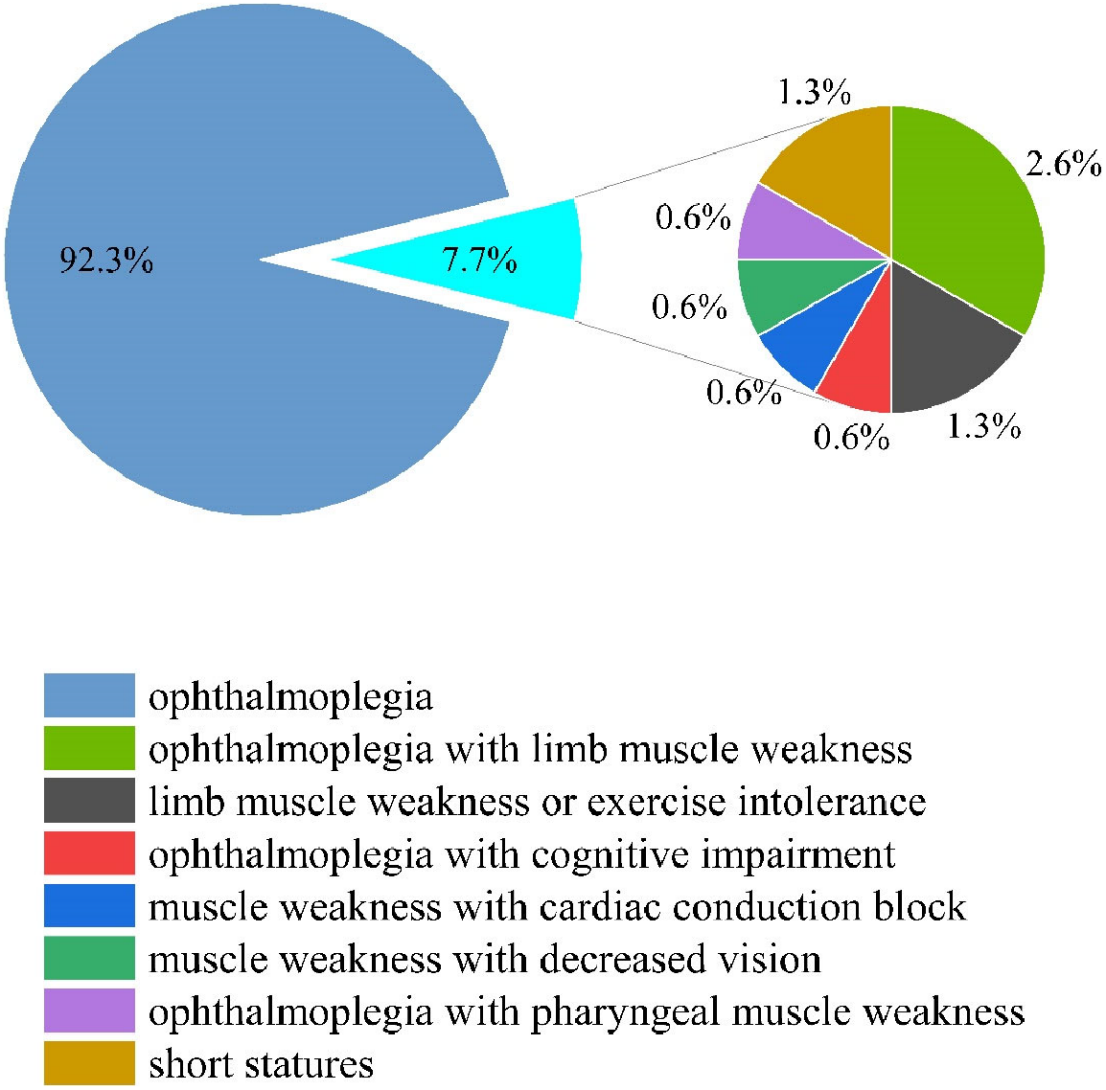
Initial symptoms of 155 patients.

In total, 155 patients were enrolled. All patients were sporadic. Among the patients, 137 (88.4%) patients had CPEO while the remaining 18 (11.6%) had KSS. Eighty‐four (54.2%) patients were female and 71 (45.8%) were male. The majority (92.3%) of patients started with ophthalmoplegia (146/155), 1.3% with limb muscle weakness or exercise intolerance (2/155), 1.3% with short stature, and 2.6% with ophthalmoplegia with limb muscle weakness (4/155). In addition, a small number of patients presented with initial symptoms that included cognitive impairment, cardiac conduction block, decreased vision, and pharyngeal muscle weakness. Details are shown in Figure 1.

Among all the patients, the onset ages were 18.92 ± 9.26 years, and the diagnosis was made at 28.79 ± 12.50 years. The mean onset age of CPEO was 20.15 ± 9.06 years (range: 4–55), while that of KSS was 9.61 ± 4.12 years (range: 2–16), and the difference was statistically significant (*p* < 0.001). The area under the curve (AUC) for discriminating CPEO and KSS by age of onset was 0.874, with a maximum Youden index of 0.594, corresponding to an optimal cut‐off point of 15.5 years.

Among the patients enrolled, 45.8% (71/155) had only ocular symptoms and were classified as pure CPEO; 42.6% (66/155) had clinical manifestations beyond the eye and were classified as CPEO plus. Additionally, 18 patients had KSS, accounting for 11.6% (18/155). Although there was a significant difference in ages of onset between the KSS, CPEO plus, and pure CPEO groups (*p* < 0.001), post hoc two‐by‐two comparisons using Bonferroni's method showed that the difference mainly arose between KSS and the two different subgroups of CPEO. There was no significant difference in the onset ages between pure CPEO and CPEO plus.

In the CPEO group, 97.08% (133/137) of patients had ptosis and 62.77% (86/137) had oculomotor restriction, while diplopia was less common (21.90%, 30/137). None of the patients had retinitis pigmentosa, but 9.49% (13/137) had decreased vision. The most frequent extraocular symptom was exercise intolerance (40.15%, 55/137), which affected most of the CPEO plus patients (83.33%, 55/66). Other symptoms such as dysacusis and dysarthria were less prevalent (<15%) in the CPEO group, while cerebellar ataxia was absent. Table S1 shows the detailed clinical manifestations and other statistics.

In the KSS group, all of whom met the diagnostic criteria of KSS (Mancuso et al., 2015), the most common symptoms in the KSS group were also ocular related. Ptosis was the most common symptom observed in the KSS group (94.44%, 17/18), with a median duration of 4 years, which was significantly shorter than the CPEO group's median duration of 7 years (*p* = 0.012). Moreover, the frequency of oculomotor restriction was 88.89% (16/18) in the KSS group, which was also significantly higher than in the CPEO group (*p* = 0.034). A total of 72.22% (13/18) of the KSS patients had retinitis pigmentosa and 50.00% (9/18) had cerebellar ataxia, both of which were more prevalent than in the CPEO group (*p* < 0.001). In addition, the KSS group had a higher frequency of cognitive dysfunction (11.11% vs. 0.73%, *p* = 0.036) and dysacusis (22.22% vs. 6.57%, *p* = 0.047). Details are shown in Table S1.

Besides these symptoms, 10.94% (7/64) of the CPEO group had ECG abnormalities, with an intraventricular block in four, cardiac conduction block with unknown type in one, sinus bradycardia in one, and ST‐T changes in one patient, respectively. In the KSS group, 14 patients had ECG findings, except for one patient with sinus arrhythmia and one patient with ST‐T changes, all the other patients had cardiac conduction block with different degrees (12 patients). There was a significant difference in the frequency of cardiac conduction block between the two groups (*p* < 0.001). Detailed clinical manifestations and other statistics are shown in Table S1.

### 3.2 Pathological features

Among patients who had skeletal muscle biopsies, RRFs were detected under mGT staining in 96.53% (139/144) and RBFs under SDH staining in 98.59% (140/142). Patients who tested negative for both RRF and RBF were all in the CPEO group. All the enrolled patients had a certain percentage of COX‐negative fibers under COX staining (biopsies before 2016) or COX/SDH double staining (biopsies after 2017). The detection rate of abnormal myofibers did not significantly differ between the CPEO and KSS groups (*p* > 0.05 for all three lesions). In the CPEO group, the proportions of all three types of abnormal myofibers were higher than those in the KSS group, but only the proportion of RBFs showed a statistically significant difference between the two groups, as shown in Table 1. All three types of muscle lesions were significantly and positively correlated with each other (correlation coefficient *r* = 0.667 for RRFs and RBFs, *r* = 0.534 for RRFs and COX‐negative fibers, and *r* = 0.640 for RBFs and COX‐negative fibers, *p* < 0.001 for all pairs).

**TABLE 1:** The proportion of abnormal myofibers in each group of patients.

Pathological changes (%)		All patients	CPEO group	KSS group	*p* value
RRF	Median (IQR)	1.29 (2.29)	1.42 (2.49)	0.65 (0.95)	0.061
	Max	13.82	13.82	3.72	—
	Min	0	0	0.14	—
RBF	Median (IQR)	1.89 (3.12)	1.95 (3.22)	0.75 (1.99)	0.013^a^
	Max	14.30	14.30	6.89	—
	Min	0	0	0.15	—
COX‐negative fibers	Median (IQR)	3.83 (6.92)	4.02 (6.97)	0.74 (6.10)	0.055
	Max	49.61	49.61	28.80	—
	Min	0.24	0.24	0.42	—

^a^ The difference was statistically significant when the significance level was 0.05. Max stands for the maximum and min for the minimum.

There was a higher proportion of RRFs [0.92% (1.79%)] in patients with symptoms beyond extraocular muscles than patients without symptoms beyond extraocular muscles [1.46% (2.73%)] (*p* = 0.033). The severity of the muscle lesions did not correlate significantly with ages of onset (*p* > 0.05 for all three lesions) but did correlate with the presence or absence of some clinical symptoms. For instance, patients with exercise intolerance had higher proportions of RRFs (*p* < 0.001) and RBFs (*p* = 0.001), and similar trends were observed in patients with limb muscle weakness (*p* = 0.003 for RRFs and *p* = 0.009 for RBFs) and cerebellar ataxia (*p* = 0.034 for RRFs and *p* = 0.011 for RBFs). Moreover, patients with dysphagia or dysarthria had more severe muscle lesions (*p* < 0.05 for all three lesions in every symptom). Patients with oculomotor restriction, however, had a lower proportion of RRFs (*p* = 0.046), as detailed in Table S2.

### 3.3 Genetic features

**FIGURE 2:**
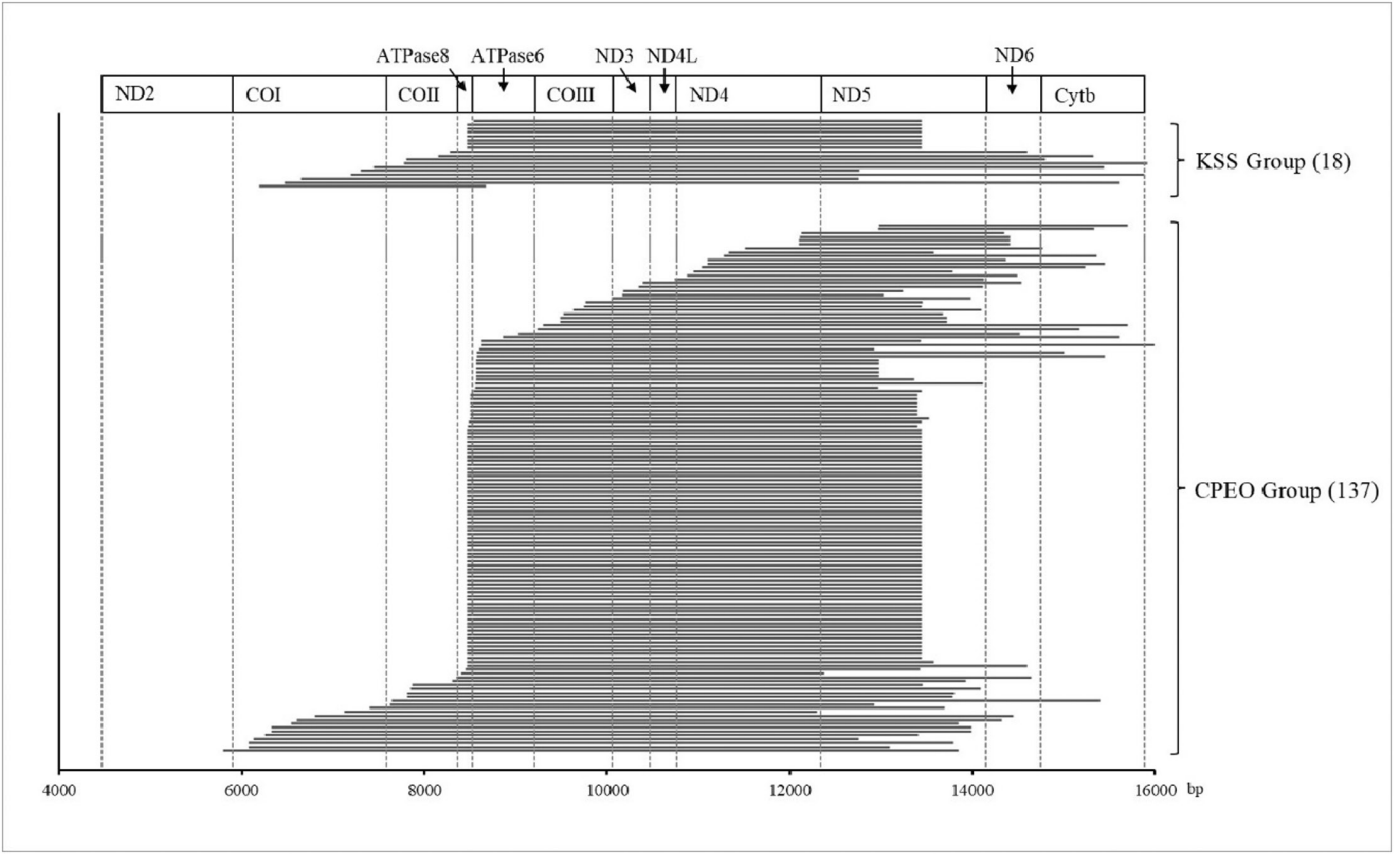
Localization of 155 deletions. ND2: NADH dehydrogenase subunit 2 [Accession (NC_012920 REGION: 4470..5511)], COI: Cytochrome *c* oxidase subunit I [Accession (NC_012920 REGION: 5904..7445)], COII: Cytochrome *c* oxidase subunit II [Accession (NC_012920 REGION: 7586..8269)], ATPase8: ATP synthase F0 subunit 8 [Accession (NC_012920 REGION: 8366..8572)], ATPase6: ATP synthase F0 subunit 6 [Accession (NC_012920 REGION: 8527..9207)], COIII: Cytochrome *c* oxidase subunit III [Accession (NC_012920 REGION: 9207..9990)], ND3: NADH dehydrogenase subunit 3 [Accession (NC_012920 REGION: 10059..10404)], ND4L: NADH dehydrogenase subunit 4L [Accession (NC_012920 REGION: 10470..10766)], ND4: NADH dehydrogenase subunit 4 [Accession (NC_012920 REGION: 10760..12137)], ND5: NADH dehydrogenase subunit 5 [Accession (NC_012920 REGION: 12337..14148)], ND6: NADH dehydrogenase subunit 6 [Accession (NC_012920 REGION: complement(14149..14673))], Cytb: Cytochrome b [Accession (NC_012920 REGION: 14747..15887)]. These accession numbers were all version NC_012920.1 The accession number and version number of genes were found in the GenBank database.

A total of 155 patients with single large mtDNA deletion were identified. The size of the deleted fragment ranged from 2225 to 9131 bp, with an average of 5108.06 ± 1266.33 bp. The starting point of these deletions was located between nucleotide (nt) 5802 and nt 12,973, and the endpoint was located between nt 8669 and nt 16,388, all on the long arc of the mtDNA (Figure 2). We identified a total of 78 mtDNA deletion types, as detailed in Table S3. Only 11 of these deletion patterns had been reported in previous studies after comparing them with the MITOMAP database (Lott et al., 2013). Of all the detected deletions, the vast majority (93.59%, 73/78) were detected in only one patient. Three deletion types were detected at frequencies higher than 3%: the canonical deletion (m.8470_13446del) in 43.20% (67/155); m.8502_13402del in 3.90% (6/155); and m.8569_12976del in 3.20% (5/155). The latter two deletions could not be found in the MITOMAP database. A total of 65.80% (102/155) of all samples contained duplicated sequences at the beginning and end of the missing sequences, ranging from 2 to 13 bp in size. The most common type of the repetitive sequence size was 13 bp, accounting for 45.20% (70/155), which was mostly present in the m.8470_13446del. In addition, repetitive sequences of 8 bp in size accounted for 5.80% (9/155), of which 55.60% (5/9) were detected in patients with m.8569_12976del. The detailed statistics of the deleted fragments are shown in Table S2.

### 3.4 Correlation between genotype and phenotype

**FIGURE 3:**
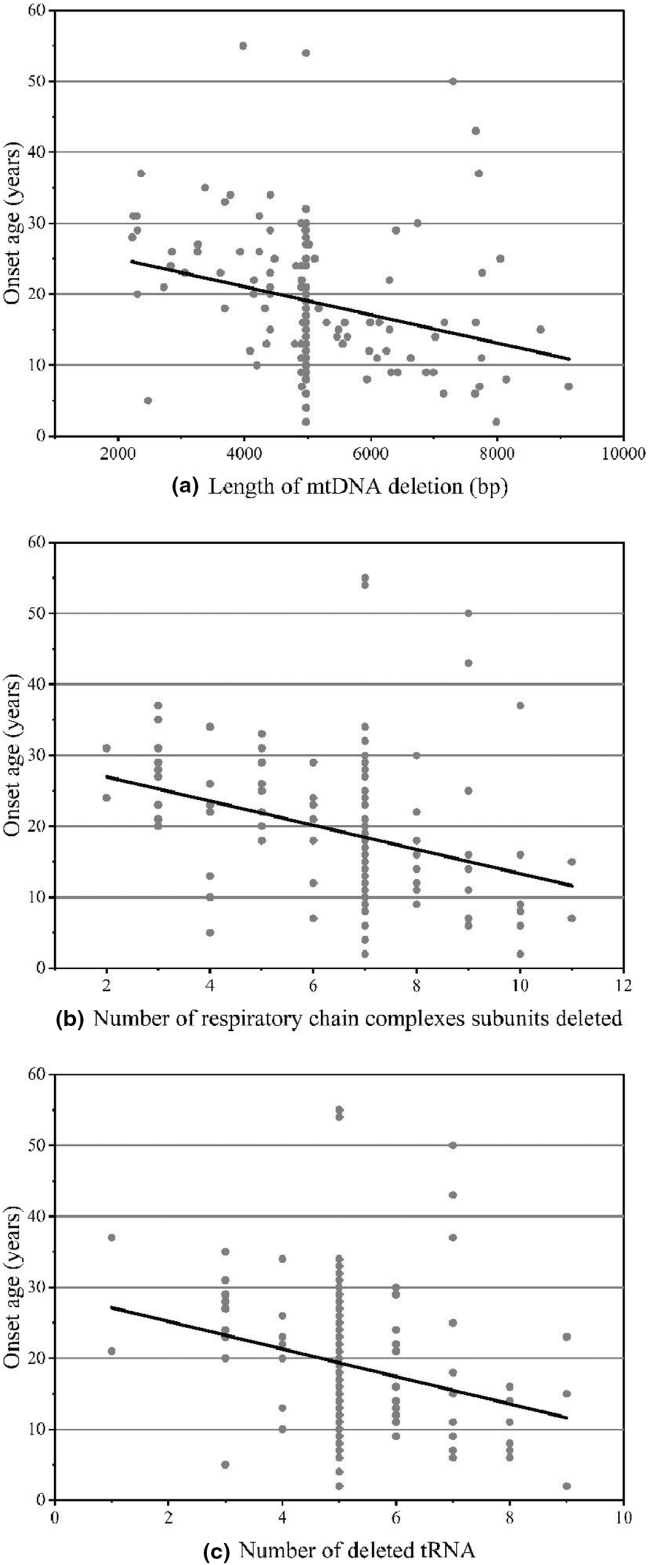
(a) Scattergram and linear regression between the length of mtDNA deletion and the onset age; (b) Scattergram and linear regression between the number of respiratory chain complex subunits deleted and the onset age; (c) Scattergram and linear regression between the number of deleted tRNAs and the onset age.

We first investigated the relationship between the deletion size, diagnosis, and onset ages. The results showed that the size of the mtDNA deletion ranged from 2225 to 8050 bp with a mean of 4989.35 ± 1153.94 bp in the CPEO group and from 2475 to 9131 bp with a mean of 6011.56 ± 1701.81 bp in the KSS group. The deletion fragment was longer in the KSS group (*p* = 0.004). In addition, there was a slight linear correlation between the onset ages and the size of the deletion fragments (*p* < 0.001, *r* = −0.369; Figure 3a).

Patients with CPEO had 6.58 ± 1.57 respiratory chain complex subunits deleted and 5.18 ± 1.13 deleted tRNAs, whereas patients with KSS had 8.06 ± 1.89 and 6.11 ± 1.64, respectively. Both the number of respiratory chain complex subunits deleted (contains ND2, ND3, ND4L, ND4, ND5, ND6, COI, COII, COIII, ATPase8, ATPase6, and Cytb) (*p* = 0.001) and the number of deleted tRNA (*p* = 0.009) were significantly higher in the KSS group than in the CPEO group. We also counted the number of deletions in the cytochrome *c* oxidase genes (MT‐CO, including MT‐CO1, MT‐CO2, and MT‐CO3) and mitochondrial cytochrome b genes (MT‐CYB), and calculated that patients with KSS had more deletions in both MT‐CO and MT‐CYB genes than patients with CPEO [median (IQR): 1.50 (1) vs. 1 (0) for MT‐CO, *p* < 0.001; 0 (1) vs. 0 (0) for MT‐CYB, *p* = 0.006]. Moreover, patients with cerebellar ataxia had more deletions in both MT‐CO and MT‐CYB genes than those without ataxia [*p* < 0.001 for MT‐CO, *p* = 0.017 for MT‐CYB]. However, no significant association was calculated between the gene deletions and other clinical symptoms.

Deletions in genes affected not only the clinical presentation but also the age of onset. The number of respiratory chain complex subunits deleted (*r* = −0.369, *p* < 0.001, Figure 3b) and the number of deleted tRNAs (*r* = −0.312, *p* < 0.001, Figure 3c) were negatively correlated with the ages of onset. We also counted the number of deletions in the MT‐CO and MT‐CYB genes and calculated that only the former was negatively correlated with the ages of onset (*p* < 0.001), while the latter showed no significant association (*p* = 0.073). Post hoc two‐by‐two comparisons using Bonferroni's method were performed to correct significance levels. The results showed that the difference in ages of onset among three deletions and one or two deletions of the MT‐CO gene was not significant. However, the comparison of the presence or absence of MT‐CO deletions and the number of MT‐CO deletions of two or fewer showed that the higher the number of deletions, the earlier the onset of the disease.

In addition, we analyzed the relationship between the genetic and the pathological features. We observed a weak negative correlation between the size of mtDNA deletion fragments and the percentage of RBFs (*p* = 0.043, *r* = −0.170), but not with RRFs or COX‐negative fibers. Similarly, the number of deleted tRNAs was weakly negatively correlated with the percentage of RRFs (*p* = 0.024, *r* = −0.188), but no association was seen between the number of respiratory chain complex subunits deleted and muscle lesions. Regarding the number of deleted genes, we observed weak negative correlations between the number of MT‐CO gene deletions and both the percentage of RRFs (*p* = 0.024, *r* = −0.189) and RBFs (*p* = 0.016, *r* = −0.201), as well as between the number of MT‐CYB gene deletions and the percentage of COX‐negative fibers (*p* = 0.015, *r* = −0.210). However, there was no significant correlation between the number of MT‐CO gene deletions and the percentage of COX‐negative fibers (*p* = 0.848), or between the number of MT‐CYB gene deletions and the percentage of RRFs (*p* = 0.209) and RBFs (*p* = 0.218).

## 4 DISCUSSION

A total of 155 patients were enrolled in this study, and the diagnosis was confirmed by combining canonical clinical presentations such as ptosis and ophthalmoplegia with skeletal muscle biopsy features and genetic testing (Ahmed et al., 2018). All patients had single large mtDNA deletions, with the most common deletion type being m.8470_13446del, which was consistent with previous findings on mitochondrial encephalomyopathies (Crimi et al., 2005; Liu et al., 2006). However, the second (m.8502_13402del) and third (m.8569_12976del) common deletion types identified in this study were not reported in the MITOMAP database (Lott et al., 2013). Furthermore, novel mutation types were detected in this study and expanded the genetic spectrum of mtDNA mutations.

For mitochondrial DNA disorders, the efficacy of the diagnostic algorithm is paramount. Our study deployed a diagnostic algorithm that blended various complementary techniques, aiming to achieve both cost‐effectiveness and precision. We initiated our diagnostic approach with Southern blot or LR‐PCR to confirm the presence of single large‐scale deletions in muscle mtDNA. As the traditional gold standard, Southern blot sequencing reliably detects large deletions with mapped breakpoints. However, its complexity, time consumption, and inefficiency limit its clinical application (Chinault et al., 2009). Despite these limitations, it provides a foundational snapshot of the mtDNA landscape. To enhance granularity, we employed Sanger sequencing, targeting specific deletion breakpoints. Yet, in an era dominated by technological advancements, next‐generation sequencing (NGS) has emerged as a game‐changer. When faced with inconclusive results from traditional methods, we turned to NGS to inspect the entire mitochondrial genome. Massively parallel sequencing, a facet of NGS, has demonstrated diagnostic sensitivity and specificity comparable with Southern blotting and Sanger sequencing (Zhang et al., 2012). However, its substantial cost meant it was reserved as the definitive detection approach in our extensive cohort. It is imperative to emphasize that while each method boasts its unique strengths, their combined use in a coherent algorithm is what truly maximizes diagnostic accuracy. This integrated approach, reflected in our study, provides a comprehensive lens to understand mtDNA disorders, serving as a bridge to reconcile diverse findings across studies.

In this study, the patients were divided into two groups, CPEO and KSS, for analysis. The CPEO patients were not further subdivided based on the presence or absence of extraocular muscle manifestations. Due to the variable duration of patient follow‐up in this study and the progressive nature of the clinical manifestations of PEO, dividing the CPEO group into CPEO plus and pure CPEO based on the presence or absence of extraocular symptoms was of limited value.

The diagnostic criteria for classical KSS include age of onset under 20 years (Kearns & Sayre, 1958). However, the setting of this age criterion is somewhat arbitrary (Mancuso et al., 2015). In this study, we attempted to provide a basis for the age demarcation between KSS and CPEO by plotting a receiver operator characteristic (ROC) curve between the age of onset and disease diagnosis. Although the ROC curve was not very smooth due to sample size limitations and there might be some overfitting problems, it had a high AUC value (0.874). When the age cut‐off was 15.5 years, this index could maximize sensitivity and specificity and might be used as a reference for determining the age cut‐off between KSS and CPEO. Considering the relatively small size of these studies, future investigations in more patients are needed to validate it.

Consistent with previous studies, our study also showed that KSS had more frequent multi‐system involvement than CPEO, while sharing the most common features as ptosis and oculomotor restriction (Björkman et al., 2023; Yamashita et al., 2008). However, it was noteworthy that the involvement of other organs beyond extraocular muscles was not rare in this series of CPEO patients, including exercise intolerance, weakness of bulbar muscles, hearing loss, and cardiac conduction abnormalities. Additionally, we previously reported that CPEO patients showed cognitive impairment mainly affecting global cognition, executive functions, and language while sparing working memory, memory, and visuospatial functions exhibited less impairment (Zhang et al., 2020). Likewise, Heighton et al. confirmed the decreased leg muscle strength in CPEO patients by assessing knee extension strength quantitatively (Heighton, Brady, Newman, et al., 2019), which was consistent with our findings. A retrospective study of 40 patients with late‐onset CPEO by Pfeffer et al. revealed that gastrointestinal dysfunction, migraine headache, and length‐dependent axonal polyneuropathy were more common than expected (Pfeffer et al., 2011). Limited by the sample size, this study did not provide a comprehensive report on the clinical manifestations of KSS patients. The enrolled KSS patients did not present symptoms such as dysarthria, gastrointestinal issues, and peripheral neuropathy (refer to Table S1 for more information). Therefore, long‐term follow‐up and serial clinical assessment in CPEO patients are necessary to obtain detailed data on the prevalence and evolution age of various manifestations over time.

Regarding the genotype–phenotype correlation, our observations revealed that KSS patients had longer mtDNA deletions when juxtaposed with CPEO patients, which also resulted in a higher number of deletions of the respiratory chain complex subunits as well as tRNA. We also perceived that the onset ages of the disease were negatively correlated with both the deletion size and the number of the respiratory chain complex subunits as well as tRNA deletions, albeit with a weak correlation. Previous studies showed inconsistent results on this point. Some studies reported a correlation between the size of mtDNA deletions in skeletal muscle cells and clinical manifestations as well as onset ages (Sadikovic et al., 2010), but others only observed a correlation between the size of deletions and onset ages (López‐Gallardo et al., 2009). The differences in the findings of the above studies could be explained by heteroplasmy, an important feature of mitochondrial diseases. Since mtDNA deletions occur during different times in embryonic development in different patients, the abundance of abnormal mtDNA varies across different tissues. Additionally, the pathogenicity thresholds for the proportion of abnormal mtDNA also vary across different tissues. These differences lead to a non‐negligible uncertainty in predicting clinical manifestations using mtDNA test results alone. Furthermore, the skeletal muscle mutant mtDNA heteroplasmy could also be an important predictor of disease progression and severity in the PEO spectrum (Mancuso et al., 2015). However, it was also shown that heteroplasmy was only mildly associated with the age of onset (Yamashita et al., 2008), suggesting that the above speculation still needs further confirmation in multicenter studies with larger samples. Unfortunately, the data on mtDNA mutation load were not available because patients in this study only underwent diagnostic genetic testing, which did not include the assessment of heteroplasmy.

In addition to genetic testing, muscle pathology biopsy plays an important role in the diagnosis of mitochondrial PEO. The typical myopathological changes include RRFs, RBFs, and COX‐negative fibers (Schon et al., 1997). RRF is caused by structural and functional abnormalities in mitochondria, as well as proliferation and aggregation, so mGT staining reflecting the aggregation of mitochondria can detect RRFs. RBF is also a common muscle pathology manifestation in mitochondrial diseases (Reichmann et al., 1996). COX is the last component of the respiratory chain, which is encoded by both mtDNA and nDNA (Rak et al., 2016); thus, the deletion of a large mtDNA segment may cause synthesis impairments of some subunits of COX, leading to a suppression of COX activity (Tanji & Bonilla, 2006). In this study, RRFs, RBFs, and COX‐deficient myofibers all showed a high positive rate in mitochondrial PEO, with COX‐negative fibers as the most sensitive pathological indicator.

Previous studies quantified the relationship between the degree of COX deficiency and mitochondrial genetics in skeletal muscles from patients with CPEO using immunofluorescence methods. They reported that muscle fibers with a higher degree of COX defects had higher levels of mtDNA deletions and higher total mtDNA copy numbers, where the COX deficiency was associated with complex‐specific protein‐encoding genes (Rocha et al., 2018). Similar findings have been reported in studies with larger samples (Grady et al., 2014). In this study, we discovered significant correlations between the deletion size, the number of missing MT‐CO and MT‐CYB genes, and some muscle lesions. This suggested that both the size and locations of deletions could affect muscle lesions to a certain extent. However, the low correlation coefficients might suggest that the association between the two was not strong or it might be an inevitable result of heteroplasmy effect. In addition, a correlation was found between muscle pathology and clinical presentations. The rule that the heavier the symptoms, the higher the proportion of aberrant myofibers was followed in most patients. Nonetheless, it was noticed that the CPEO group had a greater proportion of aberrant myofibers than the KSS group, and patients without oculomotor restriction manifested more severe muscle lesions. This outcome could be attributed to the fact that the muscle pathology biopsies' sampling sites were the biceps brachii or quadriceps femoris, and changes in these areas might not accurately reflect the severity of extraocular myopathy. However, it should be noted that normal elderly people also have COX‐negative fibers (Bua et al., 2006; Rygiel et al., 2016), and the effect of age differences on the proportion of COX‐negative fibers was not excluded in the qualitatively described muscle pathology findings.

The study had several limitations: First, because the patients in this study underwent genetic testing for diagnostic rather than scientific purposes, the testing for mtDNA heteroplasmy and mtDNA copy number was not performed, and therefore the above assessment could not be made in the analysis of clinical manifestations and genetic test results. Second, the data collection of patients enrolled was retrospective, and thus the missing clinical data bias could not be avoided. Third, in certain analyses, it was challenging to evade the small sample bias caused by the limited sample size of a specific group, and it unavoidably affected the accuracy of the findings.

In conclusion, we reported a large Chinese mitochondrial PEO cohort with single large mtDNA deletions, expanding the spectrum of mtDNA rearrangements. We observed that the majority of patients with KSS had an onset before 15.5 years of age. We also verified that the size and locations of the mtDNA deletions were useful for the demarcation between CPEO and KSS and were related to the ages of onset, but had a minor impact on the muscle lesions (RRF, RBF, and COX‐negative fibers). Moreover, we observed that the severity of the muscle lesions, besides indicating the diagnosis, was linked to clinical manifestations beyond the extraocular muscles.

## AUTHOR CONTRIBUTIONS

Zhaoxia Wang, Yue Hou, and Yang Zhao conceived the idea and designed studies. Yang Zhao, Yue Hou, Xutong Zhao, Tongling Liufu, and Meng Yu designed and carried out experiments. Yang Zhao and Victor Wei Zhang analyzed the data. Wei Zhang, Zhiying Xie, Yun Yuan, and Zhaoxia Wang contribute to the clinical and pathological diagnosis of PEO patients. Yang Zhao and Zhaoxia Wang wrote and edited the manuscript. All authors read and approved the final manuscript.

## FUNDING INFORMATION

This work was financially supported by grants from the Ministry of Science and Technology of China (2011ZX09307‐001‐07), the Beijing Municipal Science and Technology Commission (Z151100003915126), and National High‐Level Hospital Clinical Research Funding (Scientific Research Seed Fund of Peking University First Hospital) (2023SF56).

## CONFLICT OF INTEREST STATEMENT

No competing financial interest exists.

## ETHICS STATEMENT

This study was approved by the Ethics Committee of Peking University First Hospital (2019–181). Written informed consent was obtained from all participants or their legal guardians.

## Supporting information


Table S1.

Table S2.

Table S3.

## Data Availability

The data that support the findings of this study are available from the corresponding author upon reasonable request.
