# Supplementary material for: The clinical, myopathological, and genetic analysis of 155 Chinese mitochondrial ophthalmoplegia patients with mitochondrial DNA single large deletions
Source: Mol Genet Genomic Med. 2023 Nov 28;12(1):e2328. doi: 10.1002/mgg3.2328 (PMC10767604; doi:10.1002/mgg3.2328)
Supplement: Supplementary file 1 — Table S1. [file MGG3-12-e2328-s003.docx]

| Clinical manifestations | Incidence | | *P* value | Duration (years)** | | *P* value |
| --- | --- | --- | --- | --- | --- | --- |
|  | CPEO group | KSS group |  | CPEO group | KSS group |  |
| Ptosis | 97.08% (133/137) | 94.44% (17/18) | 0.465 | 7.00 (7.50) | 4.00 (4.50) | 0.012* |
| Oculomotor restriction | 62.77% (86/137) | 88.89% (16/18) | 0.034* | 0 (1) | 0 (1) | 0.263 |
| Diplopia | 21.90% (30/137) | 5.56% (1/18) | 0.126 | 0 (0) | 0 (0) | 0.212 |
| Decreased vision | 9.49% (13/137) | 72.22% (13/18) | < 0.001* | 0 (0) | 0 (0) | 0.625 |
| Pigmentary retinopathy | 0 | 72.22% (13/18) | < 0.001* | - | - | - |
| Exercise intolerance | 40.15% (55/137) | 44.44% (8/18) | 0.801 | 0 (1) | 0 (1) | 0.587 |
| Limb muscle weakness | 29.20% (40/137) | 44.44% (8/18) | 0.277 | 0 (1) | 0 (1) | 0.224 |
| Dysphagia | 11.68% (16/137) | 0 | 0.219 | - | - | - |
| Dysarthria | 14.60% (20/137) | 5.56% (1/18) | 0.470 | - | - | - |
| Dysacusis | 6.57% (9/137) | 22.22% (4/18) | 0.047* | - | - | - |
| Peripheral neuropathy | 4.38% (6/137) | 0 | > 0.999 | - | - | - |
| Diabetes mellitus | 0.73% (1/137) | 0 | > 0.999 | - | - | - |
| Dyspnea | 0.73% (1/137) | 0 | > 0.999 | - | - | - |
| Gastrointestinal symptoms | 0.73% (1/137) | 0 | > 0.999 | - | - | - |
| Cerebellar ataxia | 0 | 50.00% (9/18) | < 0.001* | - | - | - |
| Migraine | 0.73% (1/137) | 0 | > 0.999 | - | - | - |
| Cognitive impairment | 0.73% (1/137) | 11.11% (2/18) | 0.036* | - | - | - |
| Tremor | 0.73% (1/137) | 0 | > 0.999 | - | - | - |
| ECG abnormalities | 10.94% (7/64) | 100% (14/14) | < 0.001* | - | - | - |
| Cardiac conduction block | 7.81% (5/64) | 85.71% (12/14) | < 0.001* | - | - | - |

Table S1. Incidence and duration of different symptoms in patients with CPEO and KSS.

* The difference was statistically significant when the significance level was 0.05.

** The data did not obey normal distribution and were recorded using median (interquartile range).
