# Supplementary material for: The clinical, myopathological, and genetic analysis of 155 Chinese mitochondrial ophthalmoplegia patients with mitochondrial DNA single large deletions
Source: Mol Genet Genomic Med. 2023 Nov 28;12(1):e2328. doi: 10.1002/mgg3.2328 (PMC10767604; doi:10.1002/mgg3.2328)
Supplement: Supplementary file 2 — Table S2. [file MGG3-12-e2328-s002.docx]

| Pathological changes (%) | Ptosis | | *P* value | Oculomotor restriction | | *P* value | Pigmentary retinopathy | | *P* value | Exercise intolerance | | *P* value |
| --- | --- | --- | --- | --- | --- | --- | --- | --- | --- | --- | --- | --- |
|  | P | N |  | P | N |  | P | N |  | P | N |  |
| RRF | 1.30 (2.26) | 0.82 (3.34) | 0.727 | 0.98 (1.99) | 1.81 (2.19) | 0.046* | 0.65 (0.95) | 1.39 (2.50) | 0.085 | 2.09 (2.87) | 0.82 (1.53) | < 0.001* |
| RBF | 1.88 (3.24) | 1.98 (1.54) | 0.907 | 1.90 (3.15) | 1.85 (3.28) | 0.906 | 1.01 (1.97) | 1.93 (3.23) | > 0.050** | 2.81 (4.22) | 1.31 (2.76) | 0.001* |
| COX-negative fibers | 3.94 (6.92) | 1.85 (5.70) | 0.586 | 4.02 (10.27) | 3.39 (4.37) | 0.154 | 1.41 (6.51) | 4.01 (6.94) | 0.235 | 4.74 (9.45) | 3.62 (5.17) | 0.095 |
| Pathological changes (%) | Limb muscle weakness | | *P* value | Dysphagia | | *P* value | Dysarthria | | *P* value | Dysacusis | | *P* value |
|  | P | N |  | P | N |  | P | N |  | P | N |  |
| RRF | 1.98 (3.05) | 1.02 (1.90) | 0.003* | 3.93 (6.38) | 1.16 (1.96) | 0.001* | 3.76 (6.45) | 1.14 (1.88) | < 0.001* | 1.37 (2.94) | 1.25 (2.26) | 0.353 |
| RBF | 2.47 (5.61) | 1.55 (2.72) | 0.009* | 6.73 (7.46) | 1.66 (2.71) | 0.001* | 3.33 (7.68) | 1.62 (2.91) | 0.005* | 1.49 (2.99) | 1.89 (3.19) | 0.441 |
| COX-negative fibers | 3.70 (7.55) | 4.01 (6.79) | 0.734 | 9.90 (17.47) | 3.70 (6.35) | < 0.050* | 9.80 (13.56) | 3.58 (5.37) | 0.008* | 2.12 (3.99) | 4.02 (7.04) | 0.205 |
| Pathological changes (%) | Cognitive impairment | | *P* value | Peripheral neuropathy | | *P* value | Cerebellar ataxia | | *P* value | Cardiac conduction block | | *P* value |
|  | P | N |  | P | N |  | P | N |  | P | N |  |
| RRF | 0.93*** | 1.29 (2.31) | 0.642 | 0.79 (2.94) | 1.31 (2.29) | 0.576 | 0.40 (0.52) | 1.37 (2.37) | 0.034* | 0.82 (1.61) | 1.18 (2.42) | 0.316 |
| RBF | 1.16*** | 1.89 (3.13) | 0.387 | 1.63 (4.19) | 1.89 (3.12) | 0.887 | 0.46 (0.77) | 1.93 (3.18) | 0.011* | 1.76 (3.35) | 1.77 (3.49) | 0.404 |
| COX-negative fibers | 16,93**** | 3.79 (6.61) | 0.227 | 3.85 (6.27) | 3.83 (7.01) | 0.750 | 0.67 (3.99) | 4.01 (6.93) | 0.056 | 0.74 (6.28) | 4.02 (9.54) | 0.069 |

Table S2. The relationship between pathological findings and the occurrence of clinical symptoms.

“P” stands for positive, and “N” stands for negative. The data were recorded by medium (interquartile range). Some clinical manifestations had too low positive rates to allow the calculation of interquartile ranges and were not included in this table.

*The difference was statistically significant when the significance level was 0.05.

**The *P* value was between 0.050 and 0.051.

*** Quantitative COX-negative myofiber data obtained in only two case; therefore, IQR could not be calculated.

**** Quantitative COX-negative myofiber data obtained in only one case.
