# Supplementary material for: The clinical, myopathological, and genetic analysis of 155 Chinese mitochondrial ophthalmoplegia patients with mitochondrial DNA single large deletions
Source: Mol Genet Genomic Med. 2023 Nov 28;12(1):e2328. doi: 10.1002/mgg3.2328 (PMC10767604; doi:10.1002/mgg3.2328)
Supplement: Supplementary file 3 — Table S3. [file MGG3-12-e2328-s001.docx]

Table S3. The locations and implicated genes of mtDNA deletions in 155 patients enrolled.

| Patient ID | Diagnosis | Size of deleted fragments | Location of deleted fragments (red for novel deletions) | Deleted mitochondrial gene | Size of duplicated sequences | Number of deleted enzyme complex | Number of deleted tRNA | Number of deleted MT-CO genes | Number of deleted MT-CYB genes |
| --- | --- | --- | --- | --- | --- | --- | --- | --- | --- |
| 1 | CPEO | 3691 | 9763-9770:13454-13461,8bp | COⅢ, ND3, 4L, 4, 5; tRNA Gly Arg His Ser Leu | 8 | 5 | 5 | 1 | 0 |
| 2 | CPEO | 3059 | 10176:13235 | ND3, 4L, 4, 5; tRNA Arg His Ser Leu | 0 | 4 | 4 | 0 | 0 |
| 3 | KSS | 8685 | 7197-7202:15882-15887,5bp | COⅠ, COⅡ, COⅢ; ND3, 4L, 4, 5, 6; tRNA Ser Asp Lys Gly Arg His Ser Leu Glu; ATPase 8 ATPase 6; Cyt b | 6 | 11 | 9 | 3 | 1 |
| 4 | CPEO | 4977 | 8470-8482:13447-13459,13bp | COⅢ; ND3, 4L, 4, 5; tRNA Gly Arg His Ser Leu; ATPase 8 ATPase 6 | 13 | 7 | 5 | 1 | 0 |
| 5 | CPEO | 7722 | 6076-6085:13798-13807,10bp | COⅠ, COⅡ COⅢ, ND3, 4L, 4, 5;tRNA Ser Asp Lys Gly Arg His Ser Leu; ATPase 8 ATPase 6 | 10 | 9 | 8 | 3 | 0 |
| 6 | CPEO | 3979 | 8400-8412:12379-12391,13bp | COⅢ; ND3, 4L, 4, 5; tRNA Gly Arg His Ser Leu; ATPase 8 ATPase 6 | 12 | 7 | 5 | 1 | 0 |
| 7 | CPEO | 4977 | 8470-8482:13447-13459,13bp | COⅢ; ND3, 4L, 4, 5; tRNA Gly Arg His Ser Leu; ATPase 8 ATPase 6 | 13 | 7 | 5 | 1 | 0 |
| 8 | CPEO | 5554 | 8560-8565:14114-14119,6bp | COⅢ; ND3, 4L, 4, 5; tRNA Gly Arg His Ser Leu; ATPase 8 ATPase 6 | 5 | 7 | 5 | 1 | 0 |
| 9 | CPEO | 4236 | 9486-9498:13722-13734,13bp | COⅢ; ND3, 4L, 4, 5; tRNA Gly Arg His Ser Leu | 12 | 5 | 5 | 1 | 0 |
| 10 | CPEO | 4977 | 8470-8482:13447-13459,13bp | COⅢ; ND3, 4L, 4, 5; tRNA Gly Arg His Ser Leu; ATPase 8 ATPase 6 | 13 | 7 | 5 | 1 | 0 |
| 11 | CPEO | 4977 | 8470-8482:13447-13459,13bp | COⅢ; ND3, 4L, 4, 5; tRNA Gly Arg His Ser Leu; ATPase 8 ATPase 6 | 13 | 7 | 5 | 1 | 0 |
| 12 | CPEO | 5589 | 7863-7866:13452-13455,4bp | COⅡ, COⅢ; ND3, 4L, 4, 5; tRNA Lys Gly Arg His Ser Leu; ATPase 8 ATPase 6 | 3 | 8 | 6 | 2 | 0 |
| 13 | CPEO | 4977 | 8470-8482:13447-13459,13bp | COⅢ; ND3, 4L, 4, 5; tRNA Gly Arg His Ser Leu; ATPase 8 ATPase 6 | 13 | 7 | 5 | 1 | 0 |
| 14 | CPEO | 4977 | 8470-8482:13447-13459,13bp | COⅢ; ND3, 4L, 4, 5; tRNA Gly Arg His Ser Leu; ATPase 8 ATPase 6 | 13 | 7 | 5 | 1 | 0 |
| 15 | CPEO | 4977 | 8470-8482:13447-13459,13bp | COⅢ; ND3, 4L, 4, 5; tRNA Gly Arg His Ser Leu; ATPase 8 ATPase 6 | 15 | 7 | 5 | 1 | 0 |
| 16 | CPEO | 4977 | 8470-8482:13447-13459,13bp | COⅢ; ND3, 4L, 4, 5; tRNA Gly Arg His Ser Leu; ATPase 8 ATPase 6 | 13 | 7 | 5 | 1 | 0 |
| 17 | CPEO | 4977 | 8470-8482:13447-13459,13bp | COⅢ; ND3, 4L, 4, 5; tRNA Gly Arg His Ser Leu; ATPase 8 ATPase 6 | 13 | 7 | 5 | 1 | 0 |
| 18 | CPEO | 4977 | 8470-8482:13447-13459,13bp | COⅢ; ND3, 4L, 4, 5; tRNA Gly Arg His Ser Leu; ATPase 8 ATPase 6 | 13 | 7 | 5 | 1 | 0 |
| 19 | CPEO | 4977 | 8470-8482:13447-13459,13bp | COⅢ; ND3, 4L, 4, 5; tRNA Gly Arg His Ser Leu; ATPase 8 ATPase 6 | 13 | 7 | 5 | 1 | 0 |
| 20 | CPEO | 2308 | 12103-12112:14411-14420,10bp | ND4, 5, 6; tRNA His Ser Leu | 10 | 3 | 3 | 0 | 0 |
| 21 | CPEO | 5112 | 8468-8477:13580-13589,10bp | COⅢ; ND3, 4, 4L, 5; tRNA Gly Arg His Ser Leu; ATPase 8 ATpase 6 | 10 | 7 | 5 | 1 | 0 |
| 22 | CPEO | 4977 | 8470-8482:13447-13459,13bp | COⅢ; ND3, 4L, 4, 5; tRNA Gly Arg His Ser Leu; ATPase 8 ATPase 6 | 13 | 7 | 5 | 1 | 0 |
| 23 | CPEO | 4977 | 8470-8482:13447-13459,13bp | COⅢ; ND3, 4L, 4, 5; tRNA Gly Arg His Ser Leu; ATPase 8 ATPase 6 | 13 | 7 | 5 | 1 | 0 |
| 24 | CPEO | 4977 | 8470-8482:13447-13459,13bp | COⅢ; ND3, 4L, 4, 5; tRNA Gly Arg His Ser Leu; ATPase 8 ATPase 6 | 13 | 7 | 5 | 1 | 0 |
| 25 | CPEO | 4977 | 8470-8482:13447-13459,13bp | COⅢ; ND3, 4L, 4, 5; tRNA Gly Arg His Ser Leu; ATPase 8 ATPase 6 | 13 | 7 | 5 | 1 | 0 |
| 26 | CPEO | 5937 | 9233:15170 | COⅢ; ND3, 4L, 4, 5, 6; tRNA Gly Arg His Ser Leu Glu Cyt b | 0 | 7 | 5 | 1 | 1 |
| 27 | CPEO | 4977 | 8470-8482:13447-13459,13bp | COⅢ; ND3, 4L, 4, 5; tRNA Gly Arg His Ser Leu; ATPase 8 ATPase 6 | 13 | 7 | 5 | 1 | 0 |
| 28 | CPEO | 7663 | 6331-6341:13994-14004,11bp | COⅠ, COⅡ, COⅢ; ND3, 4L, 4, 5;tRNA Asp Lys Gly Arg His Ser Leu; ATPase 8 ATPase 6 | 10 | 9 | 7 | 3 | 0 |
| 29 | CPEO | 4977 | 8470-8482:13447-13459,13bp | COⅢ; ND3, 4L, 4, 5; tRNA Gly Arg His Ser Leu; ATPase 8 ATPase 6 | 13 | 7 | 5 | 1 | 0 |
| 30 | CPEO | 4977 | 8470-8482:13447-13459,13bp | COⅢ; ND3, 4L, 4, 5; tRNA Gly Arg His Ser Leu; ATPase 8 ATPase 6 | 13 | 7 | 5 | 1 | 0 |
| 31 | CPEO | 4794 | 8568-8570:13362-13364,3bp | COⅢ; ND3, 4L, 4, 5; TRNA Gly Arg His Ser Leu; ATPase 8 ATPase 6 | 2 | 7 | 5 | 1 | 0 |
| 32 | CPEO | 4977 | 8470-8482:13447-13459,13bp | COⅢ; ND3, 4L, 4, 5; tRNA Gly Arg His Ser Leu; ATPase 8 ATPase 6 | 13 | 7 | 5 | 1 | 0 |
| 33 | CPEO | 6247 | 7845:14092 | COⅡ, COⅢ; ND3, 4L, 4, 5; tRNA Lys Gly Arg His Ser Leu; ATPase 8 ATPase 6 | 0 | 8 | 6 | 2 | 0 |
| 34 | CPEO | 2834 | 10948-10954:13782-13788,7bp | ND4, 5; tRNA His Ser Leu | 7 | 2 | 3 | 0 | 0 |
| 35 | CPEO | 4977 | 8470-8482:13447-13459,13bp | COⅢ; ND3, 4L, 4, 5; tRNA Gly Arg His Ser Leu; ATPase 8 ATPase 6 | 13 | 7 | 5 | 1 | 0 |
| 36 | CPEO | 7663 | 6331-6341:13994-14004,11bp | COⅠ, COⅡ, COⅢ; ND3, 4L, 4, 5; tRNA Ser Asp Lys Gly Arg His Ser Leu; ATPase 8 ATPase 6 | 11 | 9 | 8 | 3 | 0 |
| 37 | CPEO | 7153 | 6257:13410 | COⅠ, COⅡ, COⅢ; ND3, 4L, 4, 5; tRNA Ser Asp Lys Gly Arg His Ser Leu; ATPase 8 ATPase 6 | 0 | 9 | 8 | 3 | 0 |
| 38 | CPEO | 3379 | 10745-10749:14124-14128,5bp | ND4L, 4, 5; tRNA His Ser Leu | 5 | 3 | 3 | 0 | 0 |
| 39 | CPEO | 3693 | 9748-9752:13441-13445,6bp | COⅢ; ND3, 4L, 4, 5; tRNA Gly Arg His Ser Leu | 5 | 5 | 5 | 1 | 0 |
| 40 | CPEO | 5990 | 7814-7819:13804-13809,6bp | COⅡ, COⅢ; ND3, 4L, 4, 5; tRNA Lys Gly Arg His Ser Leu; ATPase 8 ATPase 6 | 6 | 8 | 6 | 2 | 0 |
| 41 | CPEO | 6134 | 8464:14598 | COⅢ, ND3, 4L, 4, 5, 6; tRNA Gly Arg His Ser Leu; ATPase 8 ATPase 6 | 0 | 8 | 5 | 1 | 0 |
| 42 | CPEO | 3265 | 11102-11106:14367-14371,5bp | ND4, 5, 6; tRNA His Ser Leu | 5 | 3 | 3 | 0 | 0 |
| 43 | CPEO | 3934 | 10050:13984 | ND3, 4L, 4, 5; tRNA Gly Arg His Ser Leu | 0 | 4 | 5 | 0 | 0 |
| 44 | CPEO | 4145 | 10392:14537 | ND3, 4L, 4, 5, 6; tRNA Arg His Ser Leu | 0 | 5 | 4 | 0 | 0 |
| 45 | CPEO | 6743 | 8860:15603 | COⅢ; ND3, 4L, 4, 5, 6; tRNA Gly Arg His Ser Leu Glu; ATPase 6; Cyt b | 0 | 8 | 6 | 1 | 1 |
| 46 | CPEO | 4977 | 8470-8482:13447-13459,13bp | COⅢ; ND3, 4L, 4, 5; tRNA Gly Arg His Ser Leu; ATPase 8 ATPase 6 | 13 | 7 | 5 | 1 | 0 |
| 47 | CPEO | 4977 | 8470-8482:13447-13459,13bp | COⅢ; ND3, 4L, 4, 5; tRNA Gly Arg His Ser Leu; ATPase 8 ATPase 6 | 13 | 7 | 5 | 1 | 0 |
| 48 | CPEO | 4977 | 8470-8482:13447-13459,13bp | COⅢ; ND3, 4L, 4, 5; tRNA Gly Arg His Ser Leu; ATPase 8 ATPase 6 | 13 | 7 | 5 | 1 | 0 |
| 49 | CPEO | 6298 | 7399-7409:13697-13707,11bp | COⅠ, COⅡ, COⅢ; ND3, 4L; tRNA Ser Asp Lys Gly Arg; ATPase 8 ATPase 6 | 11 | 7 | 5 | 3 | 0 |
| 50 | CPEO | 5973 | 7809-7814:13782-13788,7bp | COⅡ, COⅢ; ND3, 4L, 4, 5; tRNA Lys Gly Arg His Ser Leu; ATPase 8 ATPase 6 | 7 | 8 | 6 | 2 | 0 |
| 51 | CPEO | 4977 | 8470-8482:13447-13459,13bp | COⅢ; ND3, 4L, 4, 5; tRNA Gly Arg His Ser Leu; ATPase 8 ATPase 6 | 13 | 7 | 5 | 1 | 0 |
| 52 | CPEO | 4977 | 8470-8482:13447-13459,13bp | COⅢ; ND3, 4L, 4, 5; tRNA Gly Arg His Ser Leu; ATPase 8 ATPase 6 | 13 | 7 | 5 | 1 | 0 |
| 53 | CPEO | 3258 | 11510-11517:14768-14775,8bp | ND4, 5, 6; tRNA His Ser Leu(CUN) Glu Cyt b | 8 | 4 | 4 | 0 | 1 |
| 54 | CPEO | 6634 | 6126:12760 | COⅠ, COⅡ, COⅢ; ND3, 4L, 4, 5; tRNA Ser Asp Lys Gly Arg His Ser Leu; ATPase 8 ATPase 6 | 0 | 9 | 8 | 3 | 0 |
| 55 | CPEO | 4977 | 8470-8482:13447-13459,13bp | COⅢ; ND3, 4L, 4, 5; tRNA Gly Arg His Ser Leu; ATPase 8 ATPase 6 | 13 | 7 | 5 | 1 | 0 |
| 56 | CPEO | 7752 | 7646-7653:15398-15405,7bp | COⅡ, COⅢ; ND3, 4L, 4, 5, 6; tRNA Lys Gly Arg His Ser Leu Glu; Cyt b | 8 | 8 | 7 | 2 | 1 |
| 57 | CPEO | 4977 | 8470-8482:13447-13459,13bp | COⅢ; ND3, 4L, 4, 5; tRNA Gly Arg His Ser Leu; ATPase 8 ATPase 6 | 13 | 7 | 5 | 1 | 0 |
| 58 | CPEO | 2308 | 12103-12112:14411-14420,10bp | ND4, 5, 6; tRNA His Ser Leu | 10 | 3 | 3 | 0 | 0 |
| 59 | CPEO | 4409 | 8559-8564:12968-12973,6bp | COⅢ, ND3, 4L, 4, 5; tRNA Gly Arg His Ser Leu; ATPase 8 ATPase 6 | 6 | 7 | 5 | 1 | 0 |
| 60 | CPEO | 7021 | 6073:13094 | COⅠ, COⅡ, COⅢ; ND3, 4L, 4, 5; tRNA Ser Asp Lys Gly Arg His Ser Leu; ATPase 8 ATPase 6 | 0 | 9 | 8 | 3 | 0 |
| 61 | CPEO | 4977 | 8470-8482:13447-13459,13bp | COⅢ; ND3, 4L, 4, 5; tRNA Gly Arg His Ser Leu; ATPase 8 ATPase 6 | 13 | 7 | 5 | 1 | 0 |
| 62 | CPEO | 4977 | 8470-8482:13447-13459,13bp | COⅢ; ND3, 4L, 4, 5; tRNA Gly Arg His Ser Leu; ATPase 8 ATPase 6 | 13 | 7 | 5 | 1 | 0 |
| 63 | CPEO | 3618 | 10871-10872:14489-14490,2bp | ND4, 5, 6; tRNA His Ser Leu | 2 | 3 | 3 | 0 | 0 |
| 64 | CPEO | 3775 | 10345-10352:14120-14127,8bp | ND3, 4L, 4, 5; tRNA Arg His Ser Leu | 8 | 4 | 4 | 0 | 0 |
| 65 | CPEO | 4977 | 8470-8482:13447-13459,13bp | COⅢ; ND3, 4L, 4, 5; tRNA Gly Arg His Ser Leu; ATPase 8 ATPase 6 | 13 | 7 | 5 | 1 | 0 |
| 66 | CPEO | 4977 | 8470-8482:13447-13459,13bp | COⅢ; ND3, 4L, 4, 5; tRNA Gly Arg His Ser Leu; ATPase 8 ATPase 6 | 13 | 7 | 5 | 1 | 0 |
| 67 | CPEO | 4977 | 8470-8482:13447-13459,13bp | COⅢ; ND3, 4L, 4, 5; tRNA Gly Arg His Ser Leu; ATPase 8 ATPase 6 | 13 | 7 | 5 | 1 | 0 |
| 68 | CPEO | 4977 | 8470-8482:13447-13459,13bp | COⅢ; ND3, 4L, 4, 5; tRNA Gly Arg His Ser Leu; ATPase 8 ATPase 6 | 13 | 7 | 5 | 1 | 0 |
| 69 | CPEO | 4407 | 8569-8576:12976-12983,8bp | COⅢ; ND3, 4, 4, 4L, 5; tRNA Gly Arg His Ser Leu; ATPase 6 | 8 | 7 | 7 | 1 | 0 |
| 70 | CPEO | 4977 | 8470-8482:13447-13459,13bp | COⅢ; ND3, 4L, 4, 5; tRNA Gly Arg His Ser Leu; ATPase 8 ATPase 6 | 13 | 7 | 5 | 1 | 0 |
| 71 | CPEO | 4407 | 8569-8576:12976-12983,8bp | COⅢ; ND3, 4, 4, 4L, 5; tRNA Gly Arg His Ser Leu; ATPase 6 | 8 | 7 | 5 | 1 | 0 |
| 72 | CPEO | 4977 | 8470-8482:13447-13459,13bp | COⅢ; ND3, 4L, 4, 5; tRNA Gly Arg His Ser Leu; ATPase 8 ATPase 6 | 13 | 7 | 5 | 1 | 0 |
| 73 | CPEO | 4977 | 8470-8482:13447-13459,13bp | COⅢ; ND3, 4L, 4, 5; tRNA Gly Arg His Ser Leu; ATPase 8 ATPase 6 | 13 | 7 | 5 | 1 | 0 |
| 74 | CPEO | 4977 | 8470-8482:13447-13459,13bp | COⅢ; ND3, 4L, 4, 5; tRNA Gly Arg His Ser Leu; ATPase 8 ATPase 6 | 13 | 7 | 5 | 1 | 0 |
| 75 | CPEO | 7300 | 6544-6551:13844-13851,8bp | COⅠ, Ⅱ, Ⅲ; ND3, 4L, 4, 5; tRNA Ser Asp Lys Gly Arg His Leu; ATPase 8 ATPase 6 | 8 | 9 | 7 | 3 | 0 |
| 76 | CPEO | 4977 | 8470-8482:13447-13459,13bp | COⅢ; ND3, 4L, 4, 5; tRNA Gly Arg His Ser Leu; ATPase 8 ATPase 6 | 13 | 7 | 5 | 1 | 0 |
| 77 | CPEO | 4977 | 8470-8482:13447-13459,13bp | COⅢ; ND3, 4L, 4, 5; tRNA Gly Arg His Ser Leu; ATPase 8 ATPase 6 | 13 | 7 | 5 | 1 | 0 |
| 78 | CPEO | 4237 | 9486-9497:13723-13734,12bp | COⅢ; ND3, 4L, 4, 5; tRNA Gly Arg His Ser Leu | 12 | 5 | 5 | 1 | 0 |
| 79 | CPEO | 4977 | 8470-8482:13447-13459,13bp | COⅢ; ND3, 4L, 4, 5; tRNA Gly Arg His Ser Leu; ATPase 8 ATPase 6 | 13 | 7 | 5 | 1 | 0 |
| 80 | CPEO | 4977 | 8470-8482:13447-13459,13bp | COⅢ; ND3, 4L, 4, 5; tRNA Gly Arg His Ser Leu; ATPase 8 ATPase 6 | 13 | 7 | 5 | 1 | 0 |
| 81 | CPEO | 4977 | 8470-8482:13447-13459,13bp | COⅢ; ND3, 4L, 4, 5; tRNA Gly Arg His Ser Leu; ATPase 8 ATPase 6 | 13 | 7 | 5 | 1 | 0 |
| 82 | CPEO | 4977 | 8470-8482:13447-13459,13bp | COⅢ; ND3, 4L, 4, 5; tRNA Gly Arg His Ser Leu; ATPase 8 ATPase 6 | 13 | 7 | 5 | 1 | 0 |
| 83 | CPEO | 4977 | 8470-8482:13447-13459,13bp | COⅢ; ND3, 4L, 4, 5; tRNA Gly Arg His Ser Leu; ATPase 8 ATPase 6 | 13 | 7 | 5 | 1 | 0 |
| 84 | CPEO | 4977 | 8470-8482:13447-13459,13bp | COⅢ; ND3, 4L, 4, 5; tRNA Gly Arg His Ser Leu; ATPase 8 ATPase 6 | 13 | 7 | 5 | 1 | 0 |
| 85 | CPEO | 4977 | 8470-8482:13447-13459,13bp | COⅢ; ND3, 4L, 4, 5; tRNA Gly Arg His Ser Leu; ATPase 8 ATPase 6 | 13 | 7 | 5 | 1 | 0 |
| 86 | CPEO | 2248 | 11332:13580 | ND4, 5; tRNA His Ser Leu | 0 | 2 | 3 | 0 | 0 |
| 87 | CPEO | 4977 | 8470-8482:13447-13459,13bp | COⅢ; ND3, 4L, 4, 5; tRNA Gly Arg His Ser Leu; ATPase 8 ATPase 6 | 13 | 7 | 5 | 1 | 0 |
| 88 | CPEO | 4977 | 8470-8482:13447-13459,13bp | COⅢ; ND3, 4L, 4, 5; tRNA Gly Arg His Ser Leu; ATPase 8 ATPase 6 | 13 | 7 | 5 | 1 | 0 |
| 89 | KSS | 6991 | 7808-7814:14799-14804,6bp | COⅡ, Ⅲ; ND3, 4L, 4, 5, 6; tRNA Lys Gly Arg His Ser Leu Glu; ATPase 8 ATPase 6; Cyt b | 6 | 10 | 7 | 2 | 1 |
| 90 | CPEO | 4977 | 8470-8482:13447-13459,13bp | COⅢ; ND3, 4L, 4, 5; tRNA Gly Arg His Ser Leu; ATPase 8 ATPase 6 | 13 | 7 | 5 | 1 | 0 |
| 91 | CPEO | 4977 | 8470-8482:13447-13459,13bp | COⅢ; ND3, 4L, 4, 5; tRNA Gly Arg His Ser Leu; ATPase 8 ATPase 6 | 13 | 7 | 5 | 1 | 0 |
| 92 | CPEO | 4974 | 8452-8454:13426-13429,3bp | COⅢ; ND3, 4L, 4, 5; tRNA Gly Arg His Ser Leu; ATPase 8 ATPase 6 | 3 | 7 | 5 | 1 | 0 |
| 93 | CPEO | 4977 | 8470-8482:13447-13459,13bp | COⅢ; ND3, 4L, 4, 5; tRNA Gly Arg His Ser Leu; ATPase 8 ATPase 6 | 13 | 7 | 5 | 1 | 0 |
| 94 | CPEO | 4407 | 8569-8576:12976-12983,8bp | COⅢ; ND3, 4L, 4, 5; tRNA Gly Arg His Ser Leu; ATPase 6 | 8 | 6 | 5 | 1 | 0 |
| 95 | CPEO | 4977 | 8470-8482:13447-13459,13bp | COⅢ; ND3, 4L, 4, 5; tRNA Gly Arg His Ser Leu; ATPase 8 ATPase 6 | 13 | 7 | 5 | 1 | 0 |
| 96 | CPEO | 4407 | 8569-8576:12976-12983,8bp | COⅢ; ND3, 4L, 4, 5; tRNA Gly Arg His Ser Leu; ATPase 6 | 0 | 6 | 5 | 1 | 0 |
| 97 | CPEO | 4977 | 8470-8482:13447-13459,13bp | COⅢ; ND3, 4L, 4, 5; tRNA Gly Arg His Ser Leu; ATPase 8 ATPase 6 | 13 | 7 | 5 | 1 | 0 |
| 98 | CPEO | 4407 | 8569-8576:12976-12983,8bp | COⅢ; ND3, 4L, 4, 5; tRNA Gly Arg His Ser Leu; ATPase 6 | 8 | 6 | 5 | 1 | 0 |
| 99 | CPEO | 4087 | 11275:15362 | ND4, 5, 6; tRNA His Ser Leu Glu; Cyt b | 0 | 6 | 5 | 0 | 1 |
| 100 | CPEO | 4905 | 8539:13444 | COⅢ; ND3, 4L, 4, 5; tRNA Gly Arg His Ser Leu | 0 | 4 | 4 | 1 | 0 |
| 101 | CPEO | 4977 | 8470-8482:13447-13459,13bp | COⅢ; ND3, 4L, 4, 5; tRNA Gly Arg His Ser Leu; ATPase 8 ATPase 6 | 13 | 7 | 5 | 1 | 0 |
| 102 | CPEO | 4977 | 8470-8482:13447-13459,13bp | COⅢ; ND3, 4L, 4, 5; tRNA Gly Arg His Ser Leu; ATPase 8 ATPase 6 | 13 | 7 | 5 | 1 | 0 |
| 103 | CPEO | 4977 | 8470-8482:13447-13459,13bp | COⅢ; ND3, 4L, 4, 5; tRNA Gly Arg His Ser Leu; ATPase 8 ATPase 6 | 13 | 7 | 5 | 1 | 0 |
| 104 | CPEO | 4977 | 8470-8482:13447-13459,13bp | COⅢ; ND3, 4L, 4, 5; tRNA Gly Arg His Ser Leu; ATPase 8 ATPase 6 | 13 | 7 | 5 | 1 | 0 |
| 105 | CPEO | 4977 | 8470-8482:13447-13459,13bp | COⅢ; ND3, 4L, 4, 5; tRNA Gly Arg His Ser Leu; ATPase 8 ATPase 6 | 13 | 7 | 5 | 1 | 0 |
| 106 | CPEO | 7713 | 6608:14321 | COⅠ, Ⅱ, Ⅲ; ND3, 4L, 4, 5, 6; tRNA Ser Asp Lys Gly Arg His Leu; ATPase 8 ATPase 6 | 0 | 10 | 7 | 3 | 0 |
| 107 | CPEO | 2365 | 12973:15388 | ND5, 6; tRNA Glu; Cyt b | 0 | 3 | 1 | 0 | 1 |
| 108 | CPEO | 5175 | 7127:12302 | COⅠ, Ⅱ, Ⅲ; ND3, 4L, 4; tRNA Ser Asp Lys Gly Arg His Leu; ATPase 8 ATPase 6 | 0 | 8 | 7 | 3 | 0 |
| 109 | CPEO | 6400 | 9302:15702 | COⅢ; ND3, 4L, 4; tRNA Gly Arg His Ser Leu Glu; Cyt b | 0 | 5 | 6 | 1 | 1 |
| 110 | CPEO | 5025 | 8502:13527 | COⅢ; ND3, 4L, 4, 5; tRNA Gly Arg His Ser Leu; ATPase 8 ATPase 6; | 0 | 7 | 5 | 1 | 0 |
| 111 | CPEO | 4900 | 8502:13402 | COⅢ; ND3, 4L, 4, 5; tRNA Gly Arg His Ser Leu; ATPase 8 ATPase 6 | 0 | 7 | 5 | 1 | 0 |
| 112 | CPEO | 4150 | 9527:13677 | COⅢ; ND3, 4L, 4, 5; tRNA Gly Arg His Ser Leu | 0 | 5 | 5 | 1 | 0 |
| 113 | KSS | 6100 | 6652:12752 | COⅠ, Ⅱ, Ⅲ; ND3, 4L, 4; tRNA Asp Lys Gly Arg His Ser Leu; ATPase 8 ATPase 6 | 0 | 8 | 7 | 3 | 0 |
| 114 | CPEO | 4475 | 9627:14102 | COⅢ; ND3, 4L, 4, 5; tRNA Gly Arg His Ser Leu | 0 | 5 | 5 | 1 | 0 |
| 115 | CPEO | 4325 | 8602:12927 | COⅢ; ND3, 4L, 4, 5; tRNA Gly Arg His Ser Leu; ATPase 6 | 0 | 6 | 5 | 1 | 0 |
| 116 | CPEO | 5625 | 8302:13927 | COⅢ; ND3, 4L, 4, 5; tRNA Lys Gly Arg His Ser Leu; ATPase 8 ATPase 6 | 0 | 7 | 6 | 1 | 0 |
| 117 | CPEO | 4900 | 8502:13402 | COⅢ; ND3, 4L, 4, 5; tRNA Lys Gly Arg His Ser Leu; ATPase 8 ATPase 6 | 0 | 7 | 6 | 1 | 0 |
| 118 | CPEO | 4900 | 8502:13402 | COⅢ; ND3, 4L, 4, 5; tRNA Lys Gly Arg His Ser Leu; ATPase 8 ATPase 6 | 0 | 7 | 6 | 1 | 0 |
| 119 | CPEO | 8050 | 5802:13852 | COⅢ; ND3, 4L, 4, 5, 6; tRNA Lys Gly Arg His Ser Leu Glu; ATPase 8 ATPase 6; Cyt b | 0 | 9 | 7 | 1 | 1 |
| 120 | CPEO | 5490 | 9027:14527 | COⅢ; ND3, 4L, 4, 5, 6; tRNA Gly Arg His Ser Leu; ATPase 6 | 0 | 7 | 7 | 1 | 0 |
| 121 | CPEO | 6875 | 8577:15452 | COⅢ; ND3, 4L, 4, 5; tRNA Lys Gly Arg His Ser Leu Glu; ATPase 6; Cyt b | 0 | 7 | 7 | 1 | 1 |
| 122 | CPEO | 4814 | 8626:13440 | COⅢ; ND3; 4L; 4; 5; ATPase 6; tRNA gly; arg; his; ser2; leu2 | 0 | 6 | 5 | 1 | 0 |
| 123 | CPEO | 4900 | 8502:13402 | COⅢ; ND3, 4L, 4, 5; tRNA Lys Gly Arg His Ser Leu; ATPase 8 ATPase 6 | 0 | 7 | 6 | 1 | 0 |
| 124 | CPEO | 7650 | 6802:14452 | COⅠ, Ⅱ, Ⅲ; ND3, 4L, 4, 5, 6; tRNA Ser Asp Lys Gly Arg His Leu; ATPase 8 ATPase 6 | 0 | 10 | 7 | 3 | 0 |
| 125 | CPEO | 2225 | 12127:14352 | ND4, 5, 6; tRNA His Ser Leu | 0 | 3 | 3 | 0 | 0 |
| 126 | CPEO | 4925 | 8477:13402 | COⅢ; ND3, 4L, 4, 5; tRNA Gly Arg His Ser Leu; ATPase 8 ATPase 6 | 0 | 7 | 5 | 1 | 0 |
| 127 | CPEO | 4900 | 8502:13402 | COⅢ; ND3, 4L, 4, 5; tRNA Lys Gly Arg His Ser Leu; ATPase 8 ATPase 6 | 0 | 7 | 6 | 1 | 0 |
| 128 | CPEO | 5300 | 7627:12927 | COⅡ, Ⅲ; ND3, 4L, 4, 5; tRNA Lys Gly Arg His Ser Leu; ATPase 8 ATPase 6 | 0 | 8 | 6 | 2 | 0 |
| 129 | CPEO | 6425 | 8577:15002 | COⅢ; ND3, 4L, 4, 5, 6; tRNA Gly Arg His Ser Leu Glu; ATPase 6; Cyt b | 0 | 8 | 6 | 1 | 1 |
| 130 | CPEO | 4900 | 8502:13402 | COⅢ; ND3, 4L, 4, 5; tRNA Lys Gly Arg His Ser Leu; ATPase 8 ATPase 6 | 0 | 7 | 6 | 1 | 0 |
| 131 | CPEO | 4350 | 11100:15450 | ND4, 5, 6; tRNA His Ser Leu Glu; Cyt b | 0 | 4 | 4 | 0 | 1 |
| 132 | CPEO | 7764 | 8624:16388 | COⅢ; ND3, 4L, 4, 5; tRNA Lys Gly Arg His Ser Leu Glu Thr Pro; ATPase 6; Cyt b | 0 | 7 | 9 | 1 | 1 |
| 133 | KSS | 4906 | 8538:13444 | COⅢ; ND3, 4L, 4, 5; tRNA Gly Arg His Ser Leu; ATPase 6 | 0 | 6 | 5 | 1 | 0 |
| 134 | KSS | 4977 | 8470-8482:13447-13459,13bp | COⅢ; ND3, 4L, 4, 5; tRNA Gly Arg His Ser Leu; ATPase 8 ATPase 6 | 13 | 7 | 5 | 1 | 0 |
| 135 | KSS | 9131 | 6468:15599 | COⅠ, Ⅱ, Ⅲ; ND3, 4L, 4, 5, 6; tRNA Lys Gly Arg His Ser Leu Glu; ATPase 8 ATPase 6; Cyt b | 0 | 11 | 7 | 3 | 1 |
| 136 | KSS | 2475 | 6194:8669 | COⅠ, Ⅱ; tRNA Ser Asp Lys; ATPase 8 ATPase 6 | 0 | 4 | 3 | 2 | 0 |
| 137 | KSS | 4977 | 8470-8482:13447-13459,13bp | COⅢ; ND3, 4L, 4, 5; tRNA Gly Arg His Ser Leu; ATPase 8 ATPase 6 | 13 | 7 | 5 | 1 | 0 |
| 138 | KSS | 4977 | 8470-8482:13447-13459,13bp | COⅢ; ND3, 4L, 4, 5; tRNA Gly Arg His Ser Leu; ATPase 8 ATPase 6 | 13 | 7 | 5 | 1 | 0 |
| 139 | KSS | 5465 | 7299:12764 | COⅡ, Ⅲ; ND3, 4L, 4, 5; tRNA Lys Gly Arg His Ser Leu; ATPase 8 ATPase 6 | 0 | 8 | 6 | 2 | 0 |
| 140 | KSS | 7989 | 7449:15438 | COⅡ, Ⅲ; ND3, 4L, 4, 5, 6; tRNA Asp Lys Gly Arg His Ser Leu Glu Thr; ATPase 8 ATPase 6; Cyt b | 0 | 10 | 9 | 2 | 1 |
| 141 | KSS | 4977 | 8470-8482:13447-13459,13bp | COⅢ; ND3, 4L, 4, 5; tRNA Gly Arg His Ser Leu; ATPase 8 ATPase 6 | 13 | 7 | 5 | 1 | 0 |
| 142 | CPEO | 4977 | 8470-8482:13447-13459,13bp | COⅢ; ND3, 4L, 4, 5; tRNA Gly Arg His Ser Leu; ATPase 8 ATPase 6 | 13 | 7 | 5 | 1 | 0 |
| 143 | KSS | 6321 | 8277:14598 | COⅢ; ND3, 4L, 4, 5, 6; tRNA Lys Gly Arg His Ser Leu; ATPase 8 ATPase 6 | 0 | 8 | 6 | 1 | 0 |
| 144 | KSS | 4977 | 8470-8482:13447-13459,13bp | COⅢ; ND3, 4L, 4, 5; tRNA Gly Arg His Ser Leu; ATPase 8 ATPase 6 | 13 | 7 | 5 | 1 | 0 |
| 145 | KSS | 4977 | 8470-8482:13447-13459,13bp | COⅢ; ND3, 4L, 4, 5; tRNA Gly Arg His Ser Leu; ATPase 8 ATPase 6 | 13 | 7 | 5 | 1 | 0 |
| 146 | KSS | 4977 | 8470-8482:13447-13459,13bp | COⅢ; ND3, 4L, 4, 5; tRNA Gly Arg His Ser Leu; ATPase 8 ATPase 6 | 13 | 7 | 5 | 1 | 0 |
| 147 | KSS | 7166 | 8156:15322 | COⅡ, Ⅲ; ND3, 4L, 4, 5, 6; tRNA Lys Gly Arg His Ser Leu Glu Thr; ATPase 8 ATPase 6; Cyt b | 0 | 10 | 8 | 2 | 1 |
| 148 | KSS | 8140 | 7775:15915 | COⅡ, Ⅲ; ND3, 4L, 4, 5, 6; tRNA Lys Gly Arg His Ser Leu Glu Thr; ATPase 8 ATPase 6; Cyt b | 0 | 10 | 8 | 2 | 1 |
| 149 | CPEO | 2724 | 12976:15700 | ND5; 6; Cytb; tRNA glu | 0 | 3 | 1 | 0 | 1 |
| 150 | CPEO | 4197 | 11040:15237 | ND4; 5; 6; Cytb; tRNA his; ser2; leu2; glu | 0 | 4 | 4 | 0 | 1 |
| 151 | CPEO | 2855 | 10166:13021 | ND3; 4L; 4; 5; tRNA arg; his; ser2; leu2 | 0 | 4 | 4 | 0 | 0 |
| 152 | CPEO | 2299 | 12112:14411 | ND4; 5; 6; tRNA his; ser2; leu2 | 0 | 3 | 3 | 0 | 0 |
| 153 | CPEO | 6296 | 8346:14642 | tRNA lys; gly; arg; his; ser2; leu2; ATPase 8; 6; COⅢ; ND3; 4L; 4; 5; 6 | 0 | 8 | 6 | 1 | 0 |
| 154 | CPEO | 4977 | 8470-8482:13447-13459,13bp | tRNA gly; arg; his; ser2; leu2; ATPase 8; 6; COⅢ; ND3; 4L; 4; 5 | 13 | 7 | 5 | 1 | 0 |
| 155 | CPEO | 4964 | 8482:13446,13bp | COⅢ; ND3, 4L, 4, 5; tRNA gly; arg; his; ser2; leu2; ATPase 8 ATPase 6 | 13 | 7 | 5 | 1 | 0 |
